# Regulation of Kir4.1 expression in astrocytes and astrocytic tumors: a role for interleukin-1 β

**DOI:** 10.1186/1742-2094-9-280

**Published:** 2012-12-28

**Authors:** Emanuele Zurolo, Marjolein de Groot, Anand Iyer, Jasper Anink, Erwin A van Vliet, Jan J Heimans, Jaap C Reijneveld, Jan A Gorter, Eleonora Aronica

**Affiliations:** 1 Department of (Neuro)Pathology, Academic Medical Center, University of Amsterdam, Meibergdreef 9, Amsterdam, AZ 1105, The Netherlands; 2 Department of Neurology, Academic Medical Center, University of Amsterdam, Amsterdam, The Netherlands; 3 Department of Neurology, VU University Medical Center, Amsterdam, The Netherlands; 4 Swammerdam Institute for Life Sciences, Center for Neuroscience, University of Amsterdam, Amsterdam, The Netherlands; 5 Epilepsy Institute in The Netherlands Foundation (Stichting Epilepsie Instellingen Nederland, SEIN), Heemstede, The Netherlands

**Keywords:** Epilepsy, Inflammation, Potassium channels, Interleukin-1 β, Astrocytes, Brain tumors

## Abstract

**Objective:**

Decreased expression of inwardly rectifying potassium (Kir) channels in astrocytes and glioma cells may contribute to impaired K^+^ buffering and increased propensity for seizures. Here, we evaluated the potential effect of inflammatory molecules, such as interleukin-1β (IL-1β) on Kir4.1 mRNA and protein expression.

**Methods:**

We investigated Kir4.1 (Kcnj10) and IL-1β mRNA expression in the temporal cortex in a rat model of temporal lobe epilepsy 24 h and 1 week after induction of status epilepticus (SE), using real-time PCR and western blot analysis. The U373 glioblastoma cell line and human fetal astrocytes were used to study the regulation of Kir4.1 expression in response to pro-inflammatory cytokines. Expression of Kir4.1 protein was also evaluated by means of immunohistochemistry in surgical specimens of patients with astrocytic tumors (*n* = 64), comparing the expression in tumor patients with (*n* = 38) and without epilepsy (*n* = 26).

**Results:**

Twenty-four hours after onset of SE, Kir4.1 mRNA and protein were significantly down-regulated in temporal cortex of epileptic rats. This decrease in expression was followed by a return to control level at 1 week after SE. The transient downregulation of Kir4.1 corresponded to the time of prominent upregulation of IL-1β mRNA. Expression of Kir4.1 mRNA and protein in glial cells in culture was downregulated after exposure to IL-1β. Evaluation of Kir4.1 in tumor specimens showed a significantly lower Kir4.1 expression in the specimens of patients with epilepsy compared to patients without epilepsy. This paralleled the increased presence of activated microglial cells, as well as the increased expression of IL-1β and the cytoplasmic translocation of high mobility group box 1 (HMGB1).

**Conclusions:**

Taken together, these findings indicate that alterations in expression of Kir4.1 occurring in epilepsy-associated lesions are possibly influenced by the local inflammatory environment and in particular by the inflammatory cytokine IL-1β.

## Introduction

Astrocytes, the major glial cell type of the central nervous system (CNS), are known to play a major role in normal brain signaling and their dysfunction has been shown to be critically involved in the pathogenesis of several human CNS disorders, including epilepsy (for reviews see [1,2]). One of the most important physiological functions of astrocytes is their ability to control ionic homeostasis, in particular the extracellular concentration of potassium, which influences neuronal excitability. The inwardly rectifying potassium (Kir) channel 4.1 has been identified as a key player among the potassium channels expressed in astrocytes responsible for spatial buffering [3,4]. Conditional knock-out of Kir4.1 has been shown to lead to inhibition of potassium and glutamate uptake, hyperexcitability, and seizures [5,6]. Mutations in the human Kir4.1 gene, KCNJ10, are associated with epilepsy [7] and a compromised glial potassium spatial buffering has been suggested to underlie the epilepsy phenotype [8]. In addition, alterations in expression, localization, and function of Kir4.1, have been reported in astrocytes in a number of neurological disorders, including temporal lobe epilepsy (TLE) and malignant gliomas (for review see [9]). In tissue specimens obtained from patients with epilepsy, both electrophysiological and molecular studies suggest that impaired potassium buffering and enhanced seizure susceptibility may result from reduced expression of Kir4.1 channels [10-14]. In the fluid percussion injury model in rat, a chronic dysfunction of Kir channels (with depletion of Kir4.1 immunoreactivity in processes of neocortical astrocytes) in the epileptic focus has been reported [15]. In case of astrocytic tumors, which are often associated with seizure development, mislocalization, and/or redistribution of Kir4.1, as well as changes in the expression related to the malignancy grade, have been reported [9,16-18]. In addition, attention has been focused on the role of Kir channels as critical regulators of cell division, suggesting that a loss of functional Kir4.1 may underlie the re-entry of glial cells into the cell cycle supporting gliosis and tumor development [9]. Although these observations support an important role for astrocytic Kir4.1, it remains still unclear whether the changes in Kir4.1 expression represent the cause or the consequence of epilepsy and the mechanism underlying the regulation of the expression of Kir4.1 is still matter of discussion. It has been shown that albumin uptake into astrocytes, mediated by transforming growth factor (TGF)-β receptors, produces a downregulation of Kir4.1 in these cells [19]. In spinal cord injury, the downregulation of Kir4.1 has been suggested to be dependent on the nuclear estrogen receptor signaling [20]. Moreover, recently, Kir4.1 expression has been suggested to be influenced by changes in the extracellular environment of inflammatory cytokines, such as interleukin-1β (IL-1β) [21]. Interestingly, increasing evidence supports the notion that dysregulation of the astrocyte immune-inflammatory function is a common factor predisposing or directly contributing to the generation of seizures in epilepsy of various etiologies [1,22,23].

Our major aim was to investigate the potential effect of inflammatory molecules, such as IL-1β on Kir4.1 expression using both a glioblastoma cell line and human astrocytes in culture.

The anti-inflammatory effects of the antiepileptic drug (AED) levetiracetam reported recently *in vivo* and *in vitro*[24,25], prompted us to evaluate the effect of this AED on Kir4.1 expression in cultures exposed to IL-1β. In addition, in order to detect changes in Kir4.1 expression and/or localization in tumor astrocytes and their relationship to IL-1β expression and to the tumor epileptogenicity, astrocytic tumors with and without epilepsy were studied.

## Materials and methods

### Experimental animals

Adult male Sprague–Dawley rats (Harlan CPB laboratories, Zeist, The Netherlands) weighing 300 to 500 g were used in this study which was approved by the University Animal Welfare committee. The rats were housed individually in a controlled environment (21 ± 1°C; humidity 60%; lights on 08:00 to 20:00; food and water available *ad libitum*).

### Electrode implantation and seizure induction

In order to record hippocampal EEG, a pair of insulated stainless steel electrodes (70 μm wire diameter, tips were 80 μm apart) were implanted into the left dentate gyrus (DG) under electrophysiological control as previously described [26]. A pair of stimulation electrodes was implanted in the angular bundle. Rats underwent tetanic stimulation (50 Hz) of the hippocampus in the form of a succession of trains of pulses every 13 s. Each train had a duration of 10 s and consisted of biphasic pulses (pulse duration 0.5 ms, maximal intensity 500 μA). Stimulation was stopped when the rats displayed sustained forelimb clonus and salivation for minutes, which usually occurred within 1 h. However, stimulation never lasted longer than 90 min. Differential EEG signals were amplified (10×) via a FET transistor that connected the headset to a differential amplifier (20×; CyberAmp, Axon Instruments, Burlingame, CA, USA), filtered (1 to 60 Hz), and digitized by a computer. A seizure detection program (Harmonie, Stellate Systems, Montreal, Canada) sampled the incoming signal at a frequency of 200 Hz per channel. EEG recordings were monitored also visually and screened for seizure activity. Behavior was observed during electrical stimulation and several hours thereafter. Immediately after termination of the stimulation, periodic epileptiform discharges PEDs) occurred at a frequency of 1 to 2 Hz and they were accompanied by behavioral and EEG seizures (status epilepticus).

### Rat tissue preparation for RNA isolation and western blot analysis

After decapitation, the brain was removed and dissected and the temporal cortex was cut out of the slices under a dissection microscope. Rats were decapitated in the acute phase (1 day after SE, *n* = 6) and in the latent period (1 week after SE, *n* = 6; the rats in this group did not exhibit spontaneous seizures). Age-matched rats that were implanted but not stimulated except for field potential recordings were also included (*n* = 6). All material was frozen on dry ice and stored at −80°C until use. For western blot analysis frozen samples of control (*n* = 5), 1 day post SE (*n* = 5), and 1 week after SE (*n* = 5) were homogenized in lysis buffer (as described below) and protein content was determined using the bicinchoninic acid method [27].

### Cell cultures

For experiments with astrocytes-enriched human cultures, fetal brain tissue (15 to 23 weeks of gestation) was obtained from spontaneous or medically-induced abortions with appropriate maternal written consent for brain autopsy. Resected tissue samples were collected in Dulbecco’s modified Eagle’s medium (DMEM)/HAM F10 (1:1) medium (Gibco, Life Technologies), supplemented with 50 units/mL penicillin and 50 μg/mL streptomycin and 10% fetal calf serum (FCS). Cell isolation was performed as previously described [28,29]. Briefly, after removal of meninges and blood vessels, tissue was minced and dissociated by incubation at 37°C for 20 min in a Hank’s balanced salt solution containing 2.5 mg/mL trypsin (Sigma, St. Louis, MO, USA) and 0.1 mg/mL bovine pancreatic Dnase I (Boehringer Mannheim, Germany). Tissue was triturated and washed with DMEM/HAM F10 medium, supplemented with 50 units/mL penicillin and 50 μg/mL streptomycin and 10% FCS. Cell suspension (containing approximately 0.5 g wet weight tissue/10 mL culture medium) was passed through a 70 μm cell sieve (Becton Dickinson, USA) and plated into 25 cm^2^ flasks (Falcon, Lincoln Park, NJ, USA) and maintained in a 5% CO_2_ incubator at 37°C. After 48 h the culture medium was replaced by fresh medium and cultures were subsequently fed twice a week. Cultures reached confluence after 2 to 3 weeks. Secondary astrocyte cultures were established by trypsinizing confluent cultures and sub-plating into 6- and 24-well plates (Costar; 0.5 × 10^6^ cell/well in a 6-well plate for western blot analysis or 0.1 × 10^6^ cell/well in a 24-well plate for RNA isolation and PCR) and simultaneously into 12 mm coverslips (Sigma) in 24-well plates (Costar; 2 × 10^4^ cell/well; for immunocytochemistry). More than 98% of the cells in primary culture, as well as in the successive 12 passages, were strongly immunoreactive for the astrocytic marker GFAP and S100β. In the present study astrocytes were used for immunocytochemical analyses at passage 3 to 4. The astrocytoma cell line U373 was obtained from the American Type Culture Collection (Rockville, MD, USA); cells were cultured in DMEM/HAM F10 (1:1) supplemented with 50 units/mL penicillin, 50 μg /mL streptomycin and 10% FCS.

### Treatment of cell cultures

Human recombinant (r)IL-1β (Peprotech, NJ, USA; 10 ng/mL) was applied and maintained for 24 h before harvesting the cells for RNA isolation, western blot analysis or for immunocytochemistry. In some experiments different time periods of IL-1β exposure (ranging from 10 min to 48 h) were used and rIL-6 (10 ng/mL; Strathmann Biotec A.G., Hamburg, Germany), tumor necrosis factor α (TNFα; 1 ng/mL; Peprotech, NJ, USA) and high mobility group box 1 (HMGB1; 40nM; HMGBiotech S.r.l., Milan, Italy) alone or together with IL-1β were applied and maintained in the medium for 24 h before harvesting the cells for RNA isolation. Human IL-1receptor antagonist (IL-1Ra; 1 μg/mL; Peprotech, NJ, USA) was used to neutralize IL-1β activity (applied 1 h before IL-1β). As previously shown [29] the viability of human astrocytes in culture was not influenced by the treatments. In other experiments, cells exposed to IL-1β (for 24 h) were extensively washed with phosphate-buffered saline (PBS) and incubated up to 48 h in culture medium, before harvesting them for western blot analysis.

### Preparation of cellular extracts

Cells were harvested at 24 h after treatment and washed twice with cold PBS. The samples were homogenized in lysis buffer containing 10 mM Tris (pH 8.0), 150 mM NaCl, 10% glycerol, 1% NP-40, Na orthovanadate (10.4 mg/mL), 5 mM EDTA (pH 8.0), 5 mM NaF and protease inhibitor cocktail (Boehringer Mannheim, Germany) by incubating on ice for 15 min. The homogenates were centrifuged at 14,000 rpm for 10 min and the supernatant was used for further analysis.

### Western blot analysis

Western blot analysis was performed, as previously described [30]. For electrophoresis, equal amounts of proteins (15 to 20 μg/lane) were separated on a 10% sodium dodecylsulfate-polyacrylamide gel electrophoretic (SDS-PAGE) gel. Separated proteins were transferred to nitrocellulose paper for 90 min at 100 V, using a wet electroblotting system (BioRad, Hercules, CA, USA). Membranes were blocked for 1 h in 5% non-fat dry milk in Tris-buffered saline-Tween (TBST) (20 mM Tris, 150 mM NaCl, 0.1% Tween 20, pH 7.5). The blots were incubated overnight with the primary antibody (Kir4.1 rabbit polyclonal antibody 1:1,000 in 5% milk solution, Alomone Labs, Jerusalem, Israel).

After several washes in TBST, the membranes were incubated in TBST/5% non-fat dry milk, containing the goat anti-rabbit or rabbit anti-mouse coupled to horse radish peroxidase (1:2,500; Dako, Denmark) for 1 h. After washes in TBST, immunoreactivity was visualized using ECL PLUS western blotting detection reagent (GE Healthcare Europe, Diegen, Belgium). Expression of β-actin (monoclonal mouse, Sigma, St. Louis, MO, 1:50,000) or β-tubulin (monoclonal mouse, Sigma, St Louis, MO, 1:30,000) were used as loading control. For the quantification of the blots the band intensities were measured densitometrically using the Scion Image for Windows (beta 4.02) image-analysis software. A ratio of the band intensity of the protein of interest to that of the reference protein was used to normalize expression.

### RNA isolation and real-time quantitative PCR analysis (qPCR)

For RNA isolation, 800 μL Trizol LS Reagent (Invitrogen, Carlsbad, CA, USA) was added to 0.1 to 0.5 × 10^6^ cells. After addition of 200 μg glycogen and 200 μL chloroform, the aqueous phase was isolated using Phase Lock tubes (5 Prime GmBH, Hamburg, Germany). RNA was precipitated with isopropyl alcohol, washed with 75% ethanol and dissolved in water. The concentration and purity of RNA were determined at 260/280 nm using a nanodrop spectrophotometer (Thermo Fisher Scientific, Wilmington, DE, USA).

Five micrograms of total RNA were reverse-transcribed into cDNA using oligo dT primers. Five nmol oligo dT primers were annealed to 5 μg total RNA in a total volume of 25 μL, by incubation at 72°C for 10 min, and cooled to 4°C. Reverse transcription was performed by the addition of 25 μL RT-mix, containing: First Strand Buffer (Invitrogen-Life Technologies), 2 mM dNTPs (Pharmacia, Germany), 30 U RNAse inhibitor (Roche Applied Science, Indianapolis, IN, USA) and 400 U M-MLV reverse transcriptase (Invitrogen - Life Technologies, The Netherlands). The total reaction mix (50 μL) was incubated at 37°C for 60 min, heated to 95°C for 10 min and stored at −20°C until use.

PCR primers (Eurogentec, Belgium) were designed using the Universal Probe Library of Roche (https://www.roche-applied-science.com) on the basis of the reported mRNA sequences. For the rat we used: Kir4.1/Kcnj10 (forward: gtgacaggcaaactgcttca and reverse: gggctatcagaggctgtgtc); IL-1β (forward: aaaaatgcctcgtgctgtct; reverse: tcgttgcttgtctctccttg); and GAPDH (forward: atgactctacccacggcaag; reverse: tactcagcaccagcatcacc). For the human cell cultures we used: Kir4.1 (forward: acctcggacccaagatgac; reverse: gtatccctgggcccattag); IL-1β (forward: gcatccagctacgaatctcc reverse: gaaccagcatcttcctcagc); elongation factor 1-alpha (EF1α; forward: atccacctttgggtcgcttt; reverse: ccgcaactgtctgtctcatatcac); and hypoxanthine phosphoribosyl transferase (HPRT; forward: tggcgtcgtcgtgattagtgatg; reverse: tgtaatccagcaggtcagca). For each PCR, a mastermix was prepared on ice, containing per sample: 1 μL cDNA, 2.5 μL of FastStart Reaction Mix SYBR Green I (Roche Applied Science, Indianapolis, IN, USA), 0.4 μM of both reverse and forward primers. The final volume was adjusted to 5 μL with H_2_O (PCR grade). The LightCycler® 480 Real-Time PCR System (Roche-applied-science) was used with a 384-multiwell plate format. The cycling conditions were carried out as follows: initial denaturation at 95°C for 5 min, followed by 45 cycles of denaturation at 95°C for 15 s, annealing at 55 to 60°C for 5 s and extension at 72°C for 10 s. The fluorescent product was measured by a single acquisition mode at 72°C after each cycle. For distinguishing specific from non-specific products and primer dimers, a melting curve was obtained after amplification by holding the temperature at 65°C for 15 s followed by a gradual increase in temperature to 95°C at a rate of 2.5°C s^-1^, with the signal acquisition mode set to continuous. Quantification of data was performed using the computer program LinReg PCR in which linear regression on the Log(fluorescence) per cycle number data is applied to determine the amplification efficiency per sample [31]. The starting concentration of each specific product was divided by the starting concentration of reference genes (GAPDH, for rat material; HPRT and EF1a for the cell cultures) and this ratio was compared between patient/control groups.

### Human material

The human cases included in this study were obtained from the files of the departments of neuropathology of the Academic Medical Center (AMC, University of Amsterdam) and the VU University Medical Center (VUMC), both situated in Amsterdam and both tertiary referral centers for brain tumor patients in the Netherlands. We examined immunocytochemically 73 surgical specimens of brain tumor patients with astrocytic tumors (6 WHO grade II astrocytoma; 12 WHO grade III astrocytoma; 55 glioblastoma multiforme, GBM; Table 1). Normal-appearing control cortex/white matter was obtained at autopsy from eight adult control patients without a history of seizures or other neurological diseases. All autopsies were performed within 12 h after death. Cortical samples (cortex/white matter adjacent to the lesion with reactive changes, such as astrogliosis, but not tumor cells) of five patients with non-glial brain tumors (two meningiomas, one metastasis of carcinoma, and one lymphoma) and without refractory epilepsy were also analyzed (control cortex/surgical, Table 1). Frozen tissue from histologically normal cortex (*n* = 2) and GBM (*n* = 4) samples was used for western blot analysis and total RNA prepared from normal cortex (*n* = 6) and GBM (*n* = 8; four with epilepsy and four without epilepsy) was used for qPCR.

**Table 1:** Clinical and histopathological features

	**A II(*n*** **= 6)**	**A III(*n*** **= 12)**	**GBM(*n*** **= 55)**	**Control cortex/autopsy (*n*** **= 8)**	**Control cortex/surgical (*n*** **= 5)**
Gender (m/f)	4/2	7/5	33/22	5/3	3/2
Age (years)^a^	34 (22–43)	44.7 (33–51)	56.5 (26–76)	50 (30–72)	54 (43–75)
*Location*				-	
Frontal	5	3	18	4	2
Temporal	1	3	15	1	-
Parietal	-	-	4	5	2
Occipital	-	1	3	1	-
Thalamus	-	-	1	-	-
Parietooccipital	-	2	3	-	-
Temporoccipital	-	2	1	-	1
Temporoparietal	-	-	1	-	-
Frontotemporal	-	1	3	-	-
Frontoparietal	-	-	1	-	-
*Epilepsy*	6	7	29	-	-
*Duration epilepsy (months)*^a^	6 (4–11)	4.8 (1–9)	6.5 (1–12)	-	-

^a^Mean (range); A II, Astrocytoma WHO grade II; A III, Astrocytoma WHO grade III; GBM, Glioblastoma multiforme.

A chart review was conducted of all patients. Epilepsy was defined as the experience of one or more seizures and data regarding seizure frequency and seizure type were obtained from patient histories. We collected additional data including age, gender, tumor location, and epilepsy duration. Informed consent was obtained for the use of brain tissue and for access to medical records for research purposes. Tissue was obtained and used in a manner compliant with the Declaration of Helsinki. Two neuropathologists reviewed all cases independently and the diagnosis was confirmed according to the revised WHO classification of tumors of the central nervous system [32].

### Tissue preparation for immunocytochemistry

Tissue was fixed in 10% buffered formalin and embedded in paraffin. Paraffin-embedded tissue was sectioned at 5 μm, mounted on precoated glass slides (Star Frost, Waldemar Knittel GmbH, Brunschweig, Germany) and used for immunohistochemical staining as described below.

### Antibodies

Antibodies specific for glial fibrillary acidic protein (GFAP; polyclonal rabbit, DAKO, Glostrup, Denmark; 1:4,000; monoclonal mouse; DAKO; 1:50), vimentin (mouse clone V9; DAKO; 1:1,000), neuronal nuclear protein (NeuN; mouse clone MAB377; Chemicon, Temecula, CA, USA; 1:2,000), synaptophysin (mouse clone Sy38; DAKO; 1:200; rabbit anti-synaptophysin; DAKO; 1:200), Ki67 (mouse clone MIB-1; DAKO; 1:200), (HLA)-DP, DQ, DR (HLA-DR; mouse clone CR3/43; DAKO, Glostrup, Denmark, 1:400), MAP2 (mouse clone HM2; Sigma 1:100) and p53 (Clone DO-7 + BP53-12; Neomarkers; 1:2,000), were used in the routine immunohistochemical analysis of glial tumors. For the detection of Kir4.1, we used a polyclonal rabbit antibody (Alomone Labs, Ltd, Jerusalem, Israel; 1:100); for the detection of IL-1β a polyclonal goat antibody (sc-1250, Santa Cruz Bio., CA, USA; 1:70; [33]) and for the detection of HMGB, we used a polyclonal rabbit antibody (Pharmingen, San Diego, CA, USA; 1:100; [34]; Abcam Cambridge, UK).

### Immunohistochemistry

Paraffin-embedded sections were deparaffinized, re-hydrated, and incubated for 20 min in 0.3% H_2_O_2_ diluted in methanol to quench the endogenous peroxidase activity. Antigen retrieval was performed by incubation for 10 min at 121°C in citrate buffer (0.01 M, pH 6.0), sections were washed with phosphate-buffered saline (PBS) and incubated for 30 min in 10% normal goat serum (Harlan Sera-Lab, Loughborough, Leicestershire, UK).

Coverslips with adherent cells (U373 or fetal astrocytes) were rinsed in PBS (pH 7.4) and fixed for 15 min in 4% paraformaldehyde in PBS. After rinsing, cultures were incubated in PBS containing 10% normal goat serum for 15 min prior to the incubation with the primary antibodies.

Sections were incubated with the primary antibodies overnight at 4°C. Hereafter, sections were washed in PBS and the ready-for-use Powervision peroxidase system (Immunologic, Duiven, The Netherlands) and 3,3’-diaminobenzidine (DAB; Sigma) was used to develop the color reaction. Sections were counterstained with hematoxylin, dehydrated and coverslipped. Sections incubated without the primary antibody were essentially blank. To test the specificity of the antibody, western blot analysis of the total homogenates of human histologically normal cortex (*n* = 2) and GBM (*n* = 4) samples was performed, as described above. The number of available frozen tumor samples from patient with and without epilepsy was too small to perform meaningful statistical comparisons in subgroups and to assess whether Kir4.1 expression is more directly dependent on presence or absence of seizures or tumor type by western blot analysis.

### Evaluation of immunostaining

Semi-quantitative evaluation of immunoreactivity (IR) in tumor specimens was performed as previously [33,35] using a using a semi-quantitative scale ranging from 0 to 3 (0: -, no; 1: +/−, weak; 2: +, moderate; 3: ++, strong IR). Two representative sections per case were stained and assessed with the Kir4.1 and IL-1β antibodies. The intensity score represents the predominant staining intensity found in each specimen as averaged from the selected fields and the different sections per group. The evaluation of the IR in tumor specimens was performed in the center of the lesion, the infiltration zone was disregarded. The sections were evaluated by two independent observers blind to clinical data. In case of disagreement independent reevaluation was performed by both observers to define the final score. The approximate proportion of cells showing IR (0, <1%; 1, single to 25%; 2, 26% to 50%; 3, 51% to 75%; and 4, >75%) was also scored to give information about the relative number (‘frequency’ score) of positive cells tumor specimens. As proposed before [36,37], the product of these two values (intensity and frequency scores) was taken to give the overall score (immunoreactivity total score; IR score), shown in Tables 2 and 3. We also evaluated the IR score of HLA-DR (markers of microglia activation) in tumor tissue of patients with or without epilepsy and quantitative analysis was performed for HMGB1 in these two patient groups, as previously described [34]. Briefly, three representative adjacent non-overlapping fields of the areas of interest (A II, A III, and GBM) were captured (magnification 40×) and digitized (Leica DM5000B). We counted the total number of astroglial/tumor cells and those showing nuclear or extra-nuclear HMGB1 staining.

**Table 2:** Kir4.1 and IL-1β immunoreactivity in astrocytic tumors

**IR score**	**A II(*n*** **= 6)**	**A III(*n*** **= 12)**	**GBM (*n*** **= 55 )**	**Control cortex/autopsy (*n*** **= 8)**	**Control cortex/surgical (*n*** **= 5)**
Kir4.1	3.6 ± 0.85*	7.18 ± 0.41	5.5 ± 0.30	7.13 ± 0.36	7.03 ± 0.26
IL-1β	2.46 ± 0.67*	3.9 ± 0.9*	3.75 ± 0.45*	0	0.02 ± 0.02
HLA-DR	5.4 ± 0.77*	5.7 ± 0.80*	5.83 ± 0.35*	0.04 ± 0.02	0.03 ± 0.02

A II, Astrocytoma WHO grade II; A III, Astrocytoma WHO grade III; GBM, Glioblastoma multiforme.

Values represent the average immunoreactive score (IR) ± SEM. *P <0.05 (compared to control cortex, both autopsy and surgical samples). Kir4.1 IR score for AIII > Kir4.1 score for AII (*P* <0.05).

**Table 3:** Kir4.1 and IL-1β immunoreactivity in patients with/without epilepsy and with/without levetiracetam use

**IR score**	**With epilepsy (*n*** **= 42)**	**Without epilepsy (*n*** **= 31)**	**With levetiracetam (*n*** **= 14 )**	**Without levetiracetam (*n*** **= 28)**
Kir4.1	4.9 ± 0.36*	6.5 ± 0.37	6.8 ± 0.62**	4.5 ± 0.37
IL-1β	5.5 ± 0.49*	1.3 ± 0.17	3.9 ± 1.14**	6.3 ± 0.46
HLA-DR	5.8 ± 0.31*	3.6 ± 0.36	4.28 ± 0.15	5.2 ± 0.40

Values represent the mean immunoreactive score (IR) ± SEM. *P <0.05: significant difference compared to patients without epilepsy; **P <0.05: significant difference compared to patients without levetiracetam use.

In cell cultures (U373 and fetal astrocytes) quantitative analysis was carried out for the number of Kir4.1 immunoreactive cells. All cells were counted systematically at high magnification (×40 objective; using an ocular grid and counting 1,000 cells from two separate experiments) as positive IR (including strong or intermediate intensity of labeling) or negative. The percentage of labeled Kir4.1 was calculated based on the total number of cells.

### Statistical analysis

Statistical analysis was performed with SPSS 15.0 and Prism® (Graph Pad Software, Inc.) software for Windows. To assess differences between groups, a non-parametric Kruskal-Wallis test was performed, followed by the Mann–Whitney U test. Correlations between Kir4.1 immunostaining and different variables (histopathological diagnosis, epilepsy, the use of levetiracetam and IL-1β immunoreactivity) were assessed with the Mann–Whitney U test and the Spearman’s rank correlation test. The value of *P* <0.05 was defined as statistically significant. Multiple testing was corrected by the Bonferroni correction.

## Results

### Kir4.1 and IL-1β expression in rat temporal cortex after induction of SE

**Figure 1:**
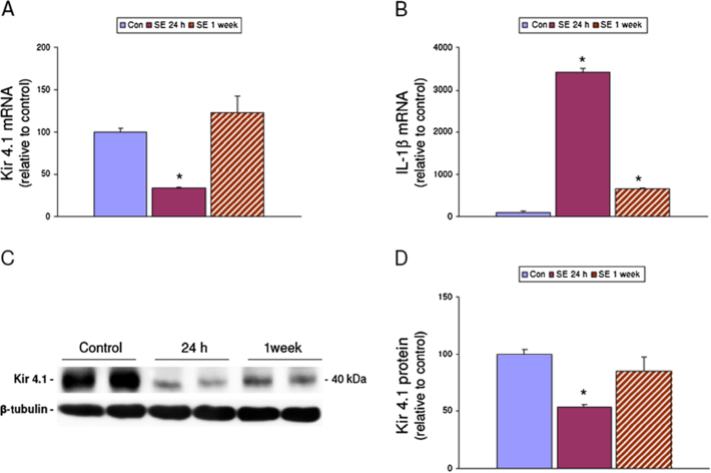
**Kir4.1 and IL-1β expression in rat temporal cortex after status epilepticus (SE).** (**A**, **B**) Quantitative real-time PCR. mRNA expression levels of Kir4.1 (**A**) and IL-1β (**B**) in the temporal cortex of control rats (*n* = 6; Con), rats at 1 week (*n* = 6), and 3 months (*n* = 6) after SE. Data represent the target gene expression normalized to the reference genes. The error bars represent SEM and * represents a *P* value <0.05. (**C**, **D**) Western blot analysis of Kir4.1. (**C**) Representative immunoblot of total homogenates from temporal cortex of controls and post SE (24 h and 1 week) rats. (**D**) Densitometric analysis: values (optical density units, O.D.) are mean ± SEM, (control, *n* = 5; 24 h post SE, *n* = 5; and 1 week post SE, *n* = 5), relative to the optical density of β-tubulin; *P <0.05, compared to controls.

To determine the temporal-spatial expression of Kir4.1 expression we performed qPCR in tissue samples of control rats and rats that were sacrificed at different time points after SE (1 day and 1 week post SE). Kir4.1 expression significantly decreased at 24 h post SE and returned toward control levels at 1 week after the onset of SE (Figure 1A). Western blot analysis of total homogenates of rat temporal cortex revealed a band at molecular weight of approximately 40 kDa which showed a significant decrease at 24 h post SE as compare to controls (Figure 1C, D). The transient prominent decrease of Kir4.1 mRNA expression following SE prompted us to evaluate whether this decrease might be related to an increased level of cytokines, such as IL-1β. Prominent IL-1β upregulation was indeed observed 24 h post SE (Figure 1B).

### Regulation of Kir4.1 expression by IL-1β in human glial cells in culture

**Figure 2:**
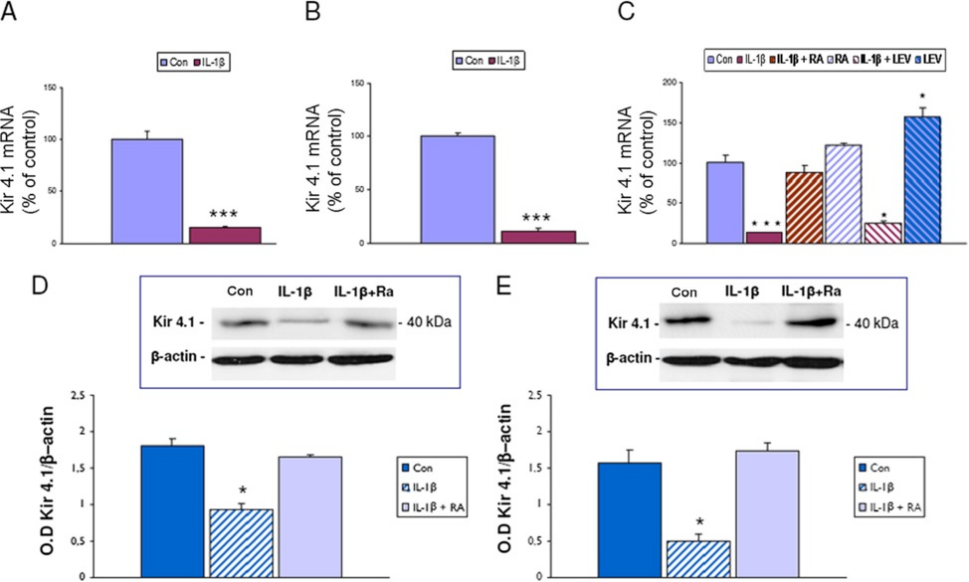
**Kir4.1 expression in U373 glioblastoma cell line and in cultured human astrocytes after exposure to IL-1β.** (**A-C**) Quantitative real-time PCR. Expression levels of Kir4.1 mRNA 24 h after exposure to IL-1β (10 ng/mL) in U373 glioblastoma cell line (**A**) and in cultured human astrocytes (**B**). (**C**) Expression levels of Kir4.1mRNA 24 h after exposure to IL-1β in U373 cell line in the presence or absence of the IL-1 β receptor antagonist (IL-1Ra; 1 μg/mL) or levetiracetam (LEV; 10 μg/mL). Data are expressed relative to the levels observed in unstimulated cells (untreated controls, Con) and are mean ± SEM from two separate experiments performed in triplicate (*P <0.05; ***P <0.0001 compared to control). (**D**, **E**). Western blot analysis of Kir4.1. Representative immunoblot of total homogenates from U373 glioblastoma cell line (**D**) and from human fetal astrocytes (**E**) untreated and treated for 24 h with 10 ng/mL IL-1β, in the presence or absence of the IL-1 β receptor antagonist (IL-1Ra; 1 μg/mL). Densitometric analysis: values (optical density units, O.D.) are mean ± SEM, relative to the optical density of β-actin; *P <0.05, compared to controls.

To address the question of whether IL-1β was involved in the modulation of Kir4.1 expression we used both human fetal astrocytes and the U373 glioblastoma cell line in culture. qPCR demonstrated that exposure to IL-1 β consistently decreased Kir4.1 expression in both cell types (Figure 2A and B). The effect of IL-1β was blocked by the IL-1Ra, a naturally occurring antagonist of the IL-1β receptor ([38]; Figure 2C). IL-1β significantly decreased Kir4.1 mRNA levels already 30 min after exposure to IL-1β (not shown) and its effect was maximal at 24 h. The downregulation of Kir4.1 mRNA could be partially reverted when IL-1β was removed and cultures were incubated for 48 h in culture medium (Kir4.1 expression (relative to control of 100%): 24 h IL-1β: 13.8% ± 2.0; 48 h after washout: 51.3% ± 2.7). In contrast, under our culture conditions we did not observe significant changes in the expression levels of Kir4.1 after exposure to IL-6 (10 ng/mL), TNFα (1 ng/mL), or HMGB1 (40nM); cytokine treatments, including IL-1β, did not influence the expression of Kir2.1, 2.3, and 3.1 mRNA (not shown).

The recently described anti-inflammatory property of the AED levetiracetam [24,25] prompted us to evaluate its effect on IL-1β–induced Kir4.1 downregulation. Exposure to levetiracetam did not affect IL-1β induced Kir4.1 downregulation. However levetiracetam treatment (for 24 h) significantly increased Kir4.1 mRNA compared to untreated cells (Figure 2C). A similar effect was observed 48 h after exposure to levetiracetam (not shown).

Western blot analysis confirmed the downregulation of Kir4.1 induced by IL-1β in U373 cells (Figure 2D) and fetal astrocytes (Figure 2E) at the protein level. No significant differences (at 24 h) in Kir4.1 protein levels were detected in either cell culture after treatment with levetiracetam (in the presence or absence of IL-1β; not shown).

**Figure 3:**
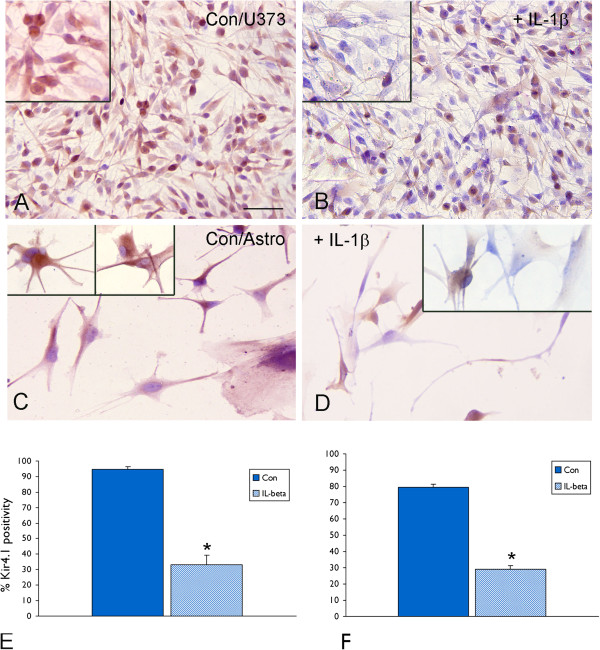
**Kir4.1 (IR) in U373 glioblastoma cell line and in cultured human astrocytes after exposure to IL-1β.** (**A**-**D**) representative photomicrographs showing Kir4.1 IR in glioma cells (U373; **A**, **B**) and cultured human astrocytes (**C**, **D**), untreated (**A** and **C**; Con) and treated (**B** and **D**) for 24 h with 10 ng/mL IL-1β; high magnifications are shown in the inserts; in U373 cells Kir4.1 was also detected in the nuclei of glial cells (insert in **A** and **B**). Scale bar in A: **A** and **B**: 80 μm; **C** and **D**: 40 μm. (**E** and **F**): percentage of Kir4.1 positivity in glioma cells (U373; **E**) and cultured human astrocytes (**F**) untreated and treated for 24 h with 10 ng/mL IL-1β. *P <0.05, compared to untreated controls (Con).

In both astrocytes-enriched human cell cultures and glioma cells incubated with IL-1β, immunocytochemistry demonstrated a lower percentage of Kir4.1-labeled cells as compared to controls (Figure 3A-F). In U373 cells (but not in fetal astrocytes), we also observed nuclear IR (Figure 3A and B) in addition to cytoplasmic Kir4.1 IR.

## Kir4.1 and IL-1β expression in human astrocytic tumors

### Patients

Table 1 summarizes the clinical and histopathological characteristics of the patients and control cases. Thirty-eight of the seventy-three tumor patients had epilepsy. The majority of the patients had secondary generalized seizures, followed by simple partial seizures. All 42 patients with epilepsy used antiepileptic drugs, 14 of them used levetiracetam before operation.

### Kir4.1 immunoreactivity

**Figure 4:**
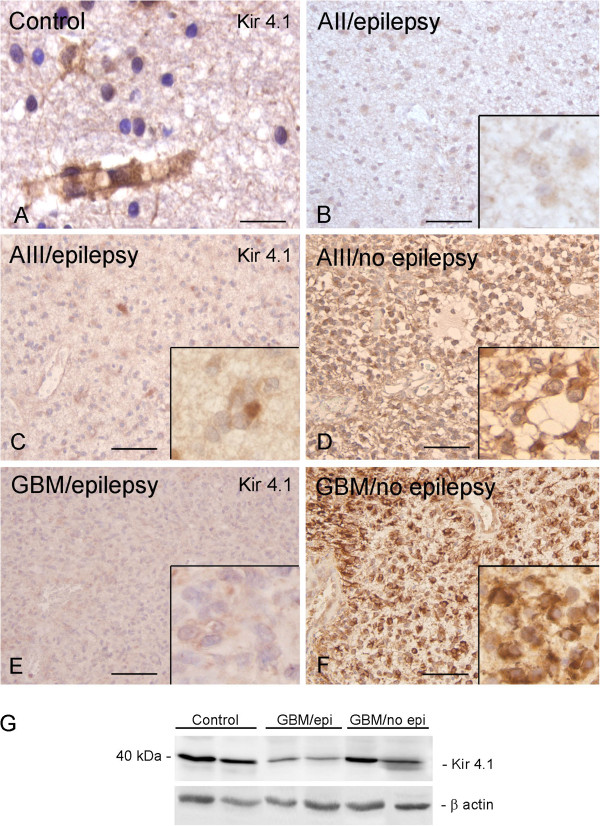
**Expression of Kir4.1 immunoreactivity (IR) in glial tumor from patients with and without epilepsy.** (**A**) Control white matter showing Kir4.1 IR in processes of perivascular astrocytes. (**B**) Astrocytoma grade II (A II); (**C**-**F**) Representative photomicrographs of Kir4.1 IR in astrocytoma grade III (A III; C and **D**) and glioblastoma multiforme (GBM; **E** and **F**) with (**C** and **E**) and without epilepsy (**D** and **F**); inserts: high magnifications, showing cytoplasmic staining, with weak IR in epilepsy-associated tumors. Sections were counterstained with hematoxylin. Scale bars: A: 40 μm; B-F: 160 μm. (**F**) Representative immunoblot of total homogenates from control cortex and GBM (with and without epilepsy) that revealed a band at a molecular weight of approximately 40 kDa.

In control tissue we did not detect obvious differences in the distribution of Kir4.1 between surgical and autopsy cortical specimens. Kir4.1 IR was detected around blood vessels (Figure 4A) as previously reported [16,17] and occasionally in the cytoplasm of astroglial cells. Astrocytoma WHO grade II and III, as well as GBM displayed mainly cytoplasmic staining in tumor cells (Figures 4B-F). The expression at perivascular endfeet membranes was less prominent and occasionally nuclear expression was observed in astrocytoma grade III and GBM (not shown). The IR score was significantly lower in astrocytoma grade II compared to control cortex, as well as astrocytoma grade III (Table 2). GBM showed variable Kir4.1 expression and the IR score was not significantly different compared to the other tumor subtypes (Table 2; Figure 4E and F). The variable Kir4.1 expression in GBM is also reflected by western blot analysis of total homogenates (Figure 4G). However, in this retrospective study, the number of frozen specimens available was too small to perform statistical comparisons in subgroups.

**Figure 5:**
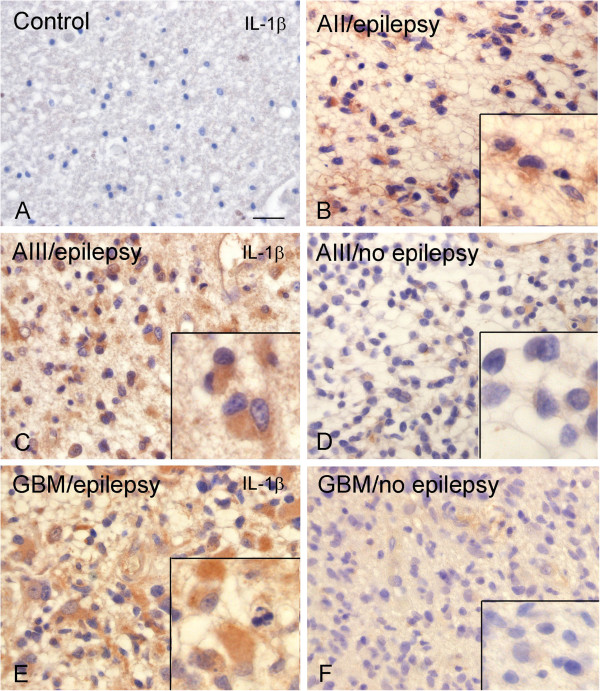
**Expression of IL-1β immunoreactivity (IR) in glial tumors from patients with and without epilepsy.** Representative photomicrographs of IL-1β IR in control white matter (**A**) astrocytoma grade II (A II; **B**, with epilepsy), astrocytoma grade III (A III; **A**-**D**) and glioblastoma multiforme (GBM; **E** and **F**) with (**C** and **E**) and without epilepsy (**D** and **F**); inserts: high magnifications, showing strong IR in epilepsy-associated tumors. Sections were counterstained with hematoxylin. A-F: scale bar in A: 80 μm.

**Figure 6:**
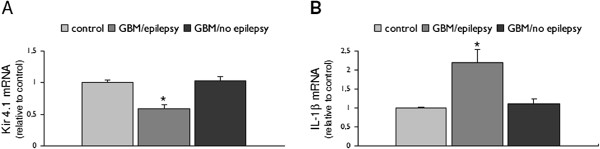
**Kir4.1 and IL-1β mRNA expression in glioblastoma multiforme (GBM) from patients with and without epilepsy.** Quantitative real-time PCR. Expression levels of Kir4.1 (**A**) and IL-1β (**B**) mRNA in GBM from patients with (*n* = 4) and without epilepsy (*n* = 4). Data are expressed relative to the levels observed in control cortex (*n* = 5) and are mean ± SEM (*P <0.05; compared to control and GBM without epilepsy).

**Figure 7:**
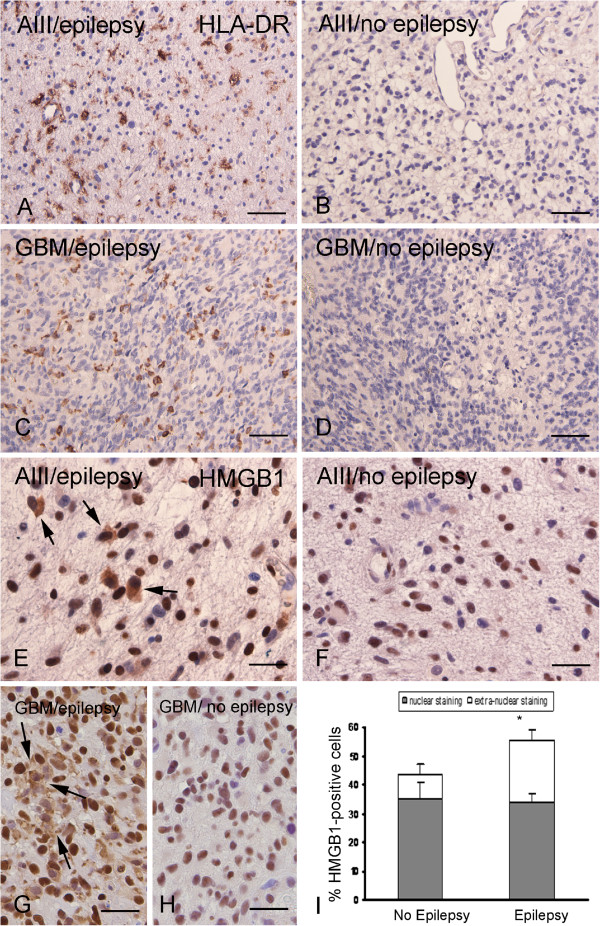
**HLA-DR and HMGB1 expression in glial tumors from patients with and without epilepsy.** (**A**-**D**) Representative photomicrographs of HLA-DR IR in astrocytoma grade III (**A** III; **A** and **B**) and glioblastoma multiforme (GBM; **C** and **D**) with (**A** and **C**) and without (**B** and **D**) epilepsy, showing immmunoreactive cells in epilepsy associated tumors. (**E**-**H**) Representative photomicrographs of HMGB1 IR in A III (**E** and **F**) and GBM (**G** and **H**) with (**E** and **G**) and without (**F** and **H**) epilepsy, showing cytoplasmic staining (arrows in **E** and **G**) in epilepsy-associated tumors. Sections were counterstained with hematoxylin. Scale bars: **A**-**C**: 160 μm; E-H: 60 μm. (**I**) Quantification bargrams of HMGB1-positive cells in tumors with and without epilepsy. Extranuclear staining in epilepsy associated tumors: *P <0.05 *vs.* tumors without epilepsy.

**Figure 8:**
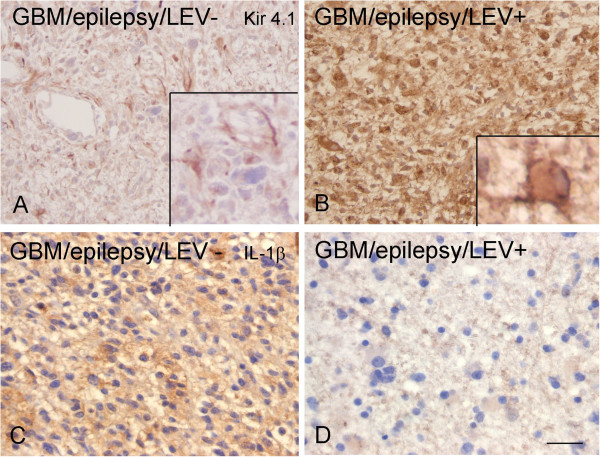
**Expression of Kir4.1 and IL-1β immunoreactivity (IR) in glial tumors from patients with epilepsy, with and without levetiracetam use.** Representative photomicrographs of Kir4.1 (**A** and **B**; high magnifications in inserts) and IL-1β (**C** and **D**) IR in glioblastoma multiforme (GBM) without (**A** and **C**) and with (**B** and **D**) levetiracetam treatment (LEV); Sections were counterstained with hematoxylin. **A**-**D:** scale bar in D: 80 μm.

### Kir4.1 expression and epilepsy

The expression and distribution of Kir4.1 IR was compared in tumor tissue of patients with astrocytoma WHO grade II, WHO grade III and GBM with or without epilepsy. A significantly lower Kir 4.1 expression was found in tumor tissue of patients with epilepsy (Figure 4B, C, E; Table 3). qPCR demonstrated lower Kir 4.1 mRNA expression in GBM with epilepsy compared to GBM without epilepsy (Figure 6A).

The number of astrocytomas grade II and grade III with and without epilepsy was too small to perform a meaningful statistical comparison between these subgroups so that we could not assess whether Kir 4.1 expression is dependent on the presence of seizures or tumor type.

### IL-1β immunoreactivity

As previously reported [33], IL-1β was under detection level in both surgical and autopsy cortical specimens of healthy controls (Figure 5A). Expression of IL-1β was detected in the different tumor subtypes in tumor cells (Figure 5B to F). The IR score for each tumor and control tissue is summarized in Table 2. The IR score was significantly higher in astrocytoma grade II, III, as well as GBM compared to control cortex. No significant differences were detected between tumor subtypes (Table 2). Astrocytoma grade II, III, as well as GBM displayed also higher IR score for HLA-DR compared to controls (Table 2).

### IL-1β expression and epilepsy

A significantly higher IL-1β expression was observed in tumor tissue of patients with epilepsy (Figure 5B, C, E; Table 3). qPCR demonstrated higher IL-1β mRNA expression in GBM with epilepsy compared to GBM without epilepsy (Figure 6B). Tumor tissue of patients with epilepsy displayed also a higher IR score for HLA-DR compared to controls (Table 3; Figure 7A-D). We also evaluated the cellular localization of HMGB1, a nuclear protein that can also act as an extracellular signal of inflammation [39-41]. We observed increased cytoplasmic translocation of HMGB1 IR in tumor tissue of patients with epilepsy compared to patients without epilepsy (Figure 7E-I).

The number of astrocytomas grade II and grade III with and without epilepsy was too small to perform a meaningful statistical comparison in these subgroups. The Spearman’s rank correlation test was applied to evaluate the relationship between Kir4.1 and IL-1β IR. A weak but significant negative correlation was observed between the Kir4.1 and the IL-1β IR score (r = −0.3663; *P* <0.05). No significant correlations were found between Kir4.1 or IL-1β IR and clinical variables such as age at surgery, age at seizure onset, and duration of epilepsy.

### Kir4.1 expression in levetiracetam-treated patients

The expression of Kir4.1 was evaluated in relation to AED regimens, in particular to levetiracetam treatment in patients with epilepsy. A significantly higher Kir4.1 IR was observed in the patients treated with levetiracetam compared to the patients who were not treated with this AED (Table 3; Figure 8A-B). In contrast a lower expression of IL-1β (Table 3; Figure 8C-D) was observed in levetiracetam-treated patients, whereas no differences were observed for HLA-DR. In addition, the seizure free interval was evaluated in levetiracetam-treated patients to assess whether Kir4.1 expression was associated with the presence of seizures and whether it was influenced by levetiracetam treatment. Of the 14 patients with epilepsy who were treated with levetiracetam, six patients were seizure free, six patients were not, and in one patient no data regarding seizure free interval was available. Kir4.1 expression was not correlated with seizure free interval in levetiracetam-treated patients.

## Discussion

The present study investigated the effect of the proinflammatory molecule IL-1β on the expression of Kir4.1, a major K^+^- inward rectifying channel in astrocytes. In addition, the expression pattern of Kir4.1 in primary human glial tumors and its relationship to seizure activity and inflammation was studied. The following observations were made: (1) in a rat model of TLE, Kir4.1 mRNA and protein were significantly downregulated in temporal cortex 24 h after onset of SE; this downregulation corresponded to the time of prominent upregulation of IL-1β; (2) IL-1β treatment reduced the expression of Kir4.1 mRNA and protein in both a glioma cell line and human astrocytes in culture; (3) Kir4.1 expression was lower in tumors with epilepsy compared to tumors without epilepsy; (4) astrocytic tumors with epilepsy displayed higher IL-1β IR compared to tumors without epilepsy; (5) among the patients with epilepsy, a significantly higher Kir4.1 IR was detected in the patients treated with levetiracetam compared to the patients who did not use this antiepileptic drug. The significance of these findings in relation to epileptogenesis in astrocytic tumors is discussed below.

### Downregulation of Kir4.1 mRNA after induction of SE parallels the increased IL-1β expression

Impaired potassium buffering and enhanced seizure susceptibility have been suggested to result from reduced expression of Kir4.1 channel in TLE ([10-14]; for review see [2]). A previous micro-array study in the electrical post-SE rat model showed that several potassium channel genes, including Kir channels were found to be downregulated 24 h after induction of SE in the CA3 region of the hippocampus [42]. The present study confirmed the downregulation of Kir4.1 mRNA at 24 h post SE in the temporal cortex. However, this decrease in expression (both mRNA and protein) recovered to control levels after the latent period. A recent study suggests a role for inflammatory cytokines, such as IL-1β, in the regulation of the expression of Kir4.1 [21]. Interestingly, experimentally-induced seizures in rodents trigger a rapid upregulation of IL-1β and its receptor ([42,43]; for review see [1,22]). IL-1β is among the best-characterized early-response inflammatory cytokines and a key mediator in the response of the brain to various forms of CNS injury (for review see [44-46]. Accordingly, in the present study, it was observed that IL-1β peaked in the temporal cortex at 1 day after SE, which corresponds to the time point of prominent reduction of Kir4.1 expression. A decrease of functional Kir channels has been shown in other pathologies associated with activation of the inflammatory response, including amyotrophic lateral sclerosis and retinopathies ([47-49]; for review see [9]). These observations suggest a role for IL-1β in the regulation of Kir4.1 mRNA expression, which was further investigated *in vitro*, using glial cells in culture.

### IL-1β treatment downregulated Kir4.1 expression human glial cells

Both U373 glioblastoma cells and human fetal astrocytes in culture expressed Kir4.1 mRNA and protein. Immunocytochemical analysis showed cytoplasmic expression of Kir4.1 in human astrocytes, whereas both cytoplasmic and nuclear expression was observed in glioma cells. This is in agreement with previous studies reporting a mislocalization of Kir channels to the nucleus in glioma cell lines [18]. In the present study, IL-1β treatment significantly decreased Kir4.1 mRNA levels in both the U373 glioma cell line and fetal astrocytes in culture. This effect (already observed at 30 min and maximal at 24 h after exposure to IL-1β) could explain the suppression of Kir4.1 mRNA expression observed after seizure-induced release of this cytokine *in vivo*. Under our experimental conditions, the effect of downregulation of Kir4.1 expression observed with IL1-β treatment could not be reproduced by other pro-inflammatory cytokines, such as IL-6 and TNFα or the toll-like receptor 4 agonist, HMGB1. The observation that IL-1Ra inhibited the effect of IL-1β on suppression of Kir4.1 is consistent with the fact that IL-1β signals through the type I IL-1β receptor. IL-1Ra is a naturally occurring antagonist of the IL-1 receptor [38], which is also regulated in response to different forms of CNS insult (for review see [1,22,50]). Thus, it is tempting to speculate that differential expression of inhibitory components of the IL-1 system and in particular local changes in the IL-1Ra/IL-1β ratio in brain, may critically contribute to the regulation of Kir4.1 expression under both physiological and pathological conditions. Moreover, the effect of IL-1β was partially reversible, with Kir4.1 levels showing partial recovery 48 h after removal of the cytokine. These observations suggest that the expression of Kir4.1 mRNA could be critically influenced by local dynamic changes in the level of IL-1β in the extracellular environment.

Recently, anti-inflammatory effects have been reported for levetiracetam [24,25], an AED frequently used to treat partial onset seizures, also in patients with brain tumors ([35,51]). In particular, treatment with this AED in neonatal rat astrocytes that were co-cultured with activated microglia or treated with IL-1β has been shown to restore impaired astrocyte membrane resting potentials via modification of inward and outward rectifier currents [25]. These studies prompted us to evaluate the effect of levetiracetam on IL-1β–induced Kir4.1 downregulation observed in human astrocytes and glioma cells. Under our experimental condition, levetiracetam was not able to counteract the downregulatory effect of IL-1β on Kir4.1 mRNA. It could be conceived that this lack of effect of levetiracetam on IL-1β treated cells is related to the dose of the cytokine used. Further experiments using different IL-1β doses in combination with levetiracetam are ongoing to address this issue. However, in the absence of IL-1β, levetiracetam positively regulated Kir4.1 mRNA expression. The potential effect of a chronic exposure to levetiracetam on IL-1β and Kir4.1 protein expression was further investigated in surgical astrocytic tumor specimens from patients treated with levetiracetam.

### Differential expression of Kir4.1 and IL-1β in astrocytic tumors

Immunocytochemical analysis showed variable Kir4.1 expression in astrocytic tumors with mainly cytoplasmic staining in tumor cells. Decrease of IR in glial processes and particularly in perivascular astrocyte endfeet was observed in both low- and high-grade gliomas, whereas nuclear expression was detected only occasionally in high-grade gliomas. Thus, the localization in the nucleus observed in glioma cell lines in culture ([18]; present study) does not represent a consistent feature of human primary glial tumor. Accordingly, nuclear localization has not been reported in other studies analyzing the expression pattern of Kir4.1 in surgical specimens of both low- and high-grade astrocytomas [16,17]. However, in agreement with our observations, Warth and colleagues [16] reported a redistribution of Kir4.1 in astrocytomas (with reduced perivascular astrocyte endfeet), suggesting a compromised buffering capacity of glial tumor cells. In our study the IR score was significantly lower in astrocytoma grade II compared to astrocytomas grade III, whereas no differences were observed compared to GBM. Tan and colleagues [17] investigated the expression of Kir4.1 mRNA and protein in astrocytic tumors and reported higher expression in high-grade astrocytic tumors compared to low-grade tumors. They suggested that activation of Kir4.1 produced intracellular alkalinization and could promote proliferation and inhibit apoptosis in the tumors [17]. In contrast, other studies suggest that the function of Kir4.1 channel is correlated with an exit from the cell cycle (for review see [9]). Thus the consequences of alterations in Kir4.1 expression on the proliferation of astrocytic tumors are still unclear and remain to be further explored. Moreover, in these previous studies [16,17], no information concerning the presence/absence of epilepsy or about the AED treatment in epileptic patients was available and considered in the evaluation of the correlation between Kir4.1 expression and pathologic tumor grade.

In the present study, we evaluated the expression of Kir4.1 and IL-1β in patients in relation to the presence or absence of epilepsy. We found a significantly lower Kir4.1 expression in tumor tissue of patients with epilepsy, which paralleled the increased expression of IL-1β. The IL-1β-mediated downregulation of Kir4.1 expression could represent an additional mechanism contributing to the pro-epileptogenic effect of this cytokine. In our study we found a significant higher IL-1β expression in tumor tissue of patients with epilepsy and a significant (although weak) negative correlation with the expression levels of Kir4.1. Interestingly, a recent study shows that minocycline treatment in the retina of diabetic rats, increases Kir4.1 levels and this effect is associated with a decrease of the levels of IL-1β [21].

Cytokine production, including also IL-1β, has been previously reported in human astrocytoma cell lines and surgical specimens of astrocytic tumors ([52-55] for review see [56]). We confirmed IL-1β expression in tumor cells, of both low- and high-grade astrocytomas, in agreement with the notion that astroglial cells represent a main source of brain IL-1β [1,57]. Accordingly, high expression of IL-1β has also been reported in tumor astrocytes in ganglioglioma, which represent a well-known cause of chronic intractable epilepsy [33]. The expression of IL-1β in tumor astrocytes may be involved in enhancing neuronal excitability in the peritumoral region (for reviews see [1,57]). A cytokine-mediated inhibition of glutamate reuptake by astrocytes may lead to increased extracellular glutamate concentrations [58,59]. Additionally, IL-1β has been shown to increase nitric oxide production and cortical glutamate release [60]. Furthermore, IL-1β may also regulate gamma-aminobutyric acid (GABA)-mediated Cl^-^ fluxes (possibly reducing inhibitory transmission) and molecular and functional interactions between IL-1β and N-methyl-D-asparte (NMDA) receptors have been recently reported (for reviews see [1,57]). Substantial experimental evidence supports the proconvulsant role of IL-1β (for review see [1,57,61]). Thus, production of IL-1β by tumor astrocyes may (through different mechanisms) contribute to the epileptogenicity of glial tumors (for review see [51]). Interestingly, the higher expression of IL-1β in tumors associated with epilepsy was linked with increased presence of activated microglial cells, as well as with the cytoplasmic translocation of HMGB1, which may contribute to amplify the inflammatory response via a signaling pathway involving the TLR4 [34]. Relocation of the nuclear protein to the cytoplasm has been shown to be induced in rat [62] and human cultured astrocytes and glioma cells by IL-1β [63]. In addition, a potential role for HMGB1 has been suggested in promoting growth and migration of human glioblastoma cells [64].

Since rapid changes in IL-1β and Kir4.1 expression are induced by seizures in experimental models (present results; for review see [1,57,61]), we cannot exclude that seizure activity may contribute to their level of expression. No significant correlation was found between Kir4.1 (or IL-1β) IR and duration of epilepsy in our cohort. However since our study does not focus on long-term epilepsy-associated tumors (LEATs; [65]), future investigations on a large cohort of LEATs are necessary to address the relationship between Kir4.1 expression and /or function and duration and/or severity of epilepsy.

As discussed above, anti-inflammatory effects have been reported for levetiracetam [24,25]. In particular, treatment with this AED (but not with valproic acid) has been shown to reduce reactive gliosis and expression levels of IL-1β in the hippocampus and the piriform cortex in chronic epileptic rats [24]. These observations prompted us to evaluate the expression levels of both IL-1β and Kir4.1 in relation to AED treatment, in particular the exposure to levetiracetam. Among the patients with epilepsy who were treated with levetiracetam, we found significantly higher expression levels of Kir4.1, and, conversely, lower expression of IL-1β, compared to patients who did not use this AED. These observations, together with previous experimental findings [24] suggest that an anti-inflammatory effect (targeting the IL-1β system) could, at least in part, contribute to the anti-epileptic effect of levetiracetam.

We acknowledge limitations to the interpretation of these results, since the cohort was relatively small to assess whether Kir4.1 expression is directly dependent on the presence or absence of seizures or on tumor type. Further quantitative analysis and *in vitro* electrophysiological studies are needed to confirm these findings and to establish their functional significance. However, this report underscores the complexity of Kir4.1 alterations in astrocytes and astrocytic tumor cells, and the potential contribution of the local inflammatory environment, involving in particular the pro-inflammatory cytokine IL-1β, in regulating the expression of Kir4.1 in epilepsy-associated lesions. Whether this mechanism could play a role in other neurological disorders (multiple sclerosis, neurodegenerative disorders) characterized by high levels of IL-1β and dysfunction of Kir4.1 [49,66,67] deserves further investigation.

## Competing interests

The authors declare that they have no competing interests.

## Authors’ contributions

The experiments in the rat model of temporal lobe epilepsy were performed by JG and EvV. Cell culture experiments were performed by EZ and AI. Immunohistochemistry, qPCR, western blot, and analysis of the data were performed by EZ, MG, AI, and JA. EZ, MG, JG, and AI helped EA in drafting and preparing the manuscript for submission. The overall experimental design was conceived and supervised by EA, JG, JR, and JH. MG, JR, and JH helped in the selection and collection of brain tissues. We are grateful to Caterina Carbonell for technical assistance. All authors read and approved the final manuscript.
